# Antiviral Activity of Svarnvir-IV Tablet Assayed for Activity Against SARS-CoV-2 In Vitro

**DOI:** 10.7759/cureus.39421

**Published:** 2023-05-24

**Authors:** K. Ramachandra Reddy, Chetan Sahni, Sanchit Sharma

**Affiliations:** 1 Department of Rasa Shastra, Faculty of Ayurveda, Institute of Medical Sciences, Banaras Hindu University, Varanasi, IND; 2 Department of Anatomy, Institute of Medical Sciences, Banaras Hindu University, Varanasi, IND; 3 Research and Development, Aimil Pharmaceuticals, Delhi, IND

**Keywords:** sars-cov-2, in vitro, virucidal activity, svarnvir-iv tablet, ayurveda

## Abstract

The coronavirus disease (COVID-19), caused by the virus SARS-CoV-2, has become a global pandemic in a very short time span. While several vaccines have been developed in the last year, specific treatments for CoV infection are still being explored. Thus, the situation highlights the need to develop safe and efficacious antiviral therapeutics. Ayurvedic *Rasayana* therapy has been traditionally used in India for its holistic healing systems and proven history of empirical use. There is emerging evidence that Ayurvedic treatment methodologies and herbal medicines may be effective strategies in combating COVID-19. The present study is aimed at evaluating the antiviral and therapeutic activity of an Ayurvedic herbomineral formulation (Svarnvir-IV tablet, 450 mg) against the SARS-CoV-2 virus *in vitro*. A cell-based assay was conducted to evaluate the cytotoxicity of the Svarnvir-IV tablets (Aimil Pharmaceuticals, Delhi, India) for the determination of virucidal activity assessment (at 2 hours) and therapeutic activity assessment (at 1 hour, 2 hours, and 4 hours). When incubated with SARS-CoV-2 virus at 0.1 multiplicity of infection (MoI) for two hours, Svarnvir-IV tablet exhibited virucidal activity against SARS-CoV-2 with an EC50 value of 0.0058 mg/ml. It also exhibited therapeutic activity when treated with cells infected with the SARS-CoV-2 virus (0.1 MoI) for 1 hour, 2 hours and 4 hours post-infection, with an EC50 value of 0.094 mg/ml, 0.023 mg/ml, and 0.05 mg/ml, respectively. The original supporting data obtained from this study, along with existing Ayurvedic traditional information, has shown encouraging results.

## Introduction

Severe acute respiratory syndrome, caused by a beta coronavirus (SARS-CoV-2), is a disease with fever, cough, shortness of breath, and dyspnea. In more severe cases, the infection can cause pneumonia, severe acute respiratory syndrome, kidney failure, and even death. Based on signs and symptoms of COVID-19 as per Ayurveda Acute Respiratory Syndrome Coronavirus 2 (SARS-CoV-2) infection, it may be compared with Ayurveda disease pathology as one of the varieties of Sannipatajajwara (wherein three doshas/genomics, Vata, Pitta, and Kapha get vitiated) associated with cough [1]. The SARS-CoV-2 virus is an encapsulated single-strand RNA virus in the Coronaviridae family that was discovered in December 2019 in China. It has now spread around the world, generating the COVID-19 pandemic, which has a high infectivity and death rate. By the beginning of April 2021, the pandemic SARS-CoV-2 had infected over 130 million individuals and killed over 2.84 million. Given the seriousness of the outbreak, experts from academia and industry are racing to develop antiviral therapies. Human coronavirus (HCV) strains (HCV-229E, HCV-OC43, HCoV-NL63, and HCoV-HKU1) are known to produce a moderate common cold with upper respiratory tract infections. In contrast, the advent of lethal human beta coronaviruses, such as the Middle East respiratory syndrome coronavirus (MERS), has been a source of concern. SARS-CoV-2 highlights the need to identify new treatment strategies for viral infections. SARS-CoV-2 is the etiological agent of COVID-19 disease, as named by the World Health Organization (WHO). The disease manifests as either an asymptomatic infection or mild to severe pneumonia [2].

As a result, efforts to swiftly develop measures to limit infection rates for the protection of vulnerable people are at an all-time high. In vitro, anti-coronavirus activity has been demonstrated in a growing variety of allopathic medications. However, scientific investigation of antiviral formulations from traditional systems like Ayurveda is yet to be explored.

Although Ayurveda is now mostly practiced in India, it had a far broader acceptance and popularity in the past, dating back to the dawn of human civilization and the Vedic period. This system has had numerous ups and downs during its long and illustrious existence. Ayurveda has lately gained international interest due to its significant utilization of plant components, processed minerals, and animal products. Ayurvedic compound formulations are basically divided into two categories:

Rasaushadhi: Medicines are prepared predominantly by processed mineral/metallic compounds (herbomineral products).

Kashtaushadhi: Medicines prepared predominantly by plant drugs.

The present study is aimed at evaluating the antiviral and virucidal (irreversible) activity by in vitro testing of an ayurvedic herbomineral formulation (Svarnvir-IV tablet, 450mg) against the SARS-CoV-2 virus to establish its potential as a formulated agent against SARS-CoV-2 infection. The herbomineral product evaluated in this study, the Svarnvir-IV tablet, belongs to the first category, i.e., Rasaushadhi. 

## Materials and methods

The study includes an evaluation of cytotoxicity on Vero cells and antiviral testing against the SARS-CoV-2 virus (virucidal activity and therapeutic activity of Svarnvir-IV tablets with a dosage of 450mg). The study was conducted by ABD-ITC Limited, with the Svarnvir-IV tablet designed by the corresponding author of the study and manufactured by Aimil Pharmaceuticals, New Delhi, India.

One of the major constituents of the Svarnvir-IV tablets is Guduchi satva. Other constituents of the Svarnvir-IV tablet are mentioned in Table 1. Guduchi satva is an extract obtained by a traditional Ayurvedic pharmaceutical procedure, and this Guduchi satva product will be similar to the water-soluble components of Guduchi. The Guduchi satva contains several phytochemical constituents/metabolites. One of the components of Guduchi (Tinosporacordifolia), i.e., Tinocordiside, was shown to inhibit the main viral protease in a molecular docking study [3]. 

**Table 1:** Ingredients of Svarnavir-IV tablet 450 mg QS: Quantum satis

Sl. No.	Ayurveda name of the ingredient in fine powder form	Part used	Botanical name	Quantity in milligrams (mg)
1.	Guduchi satva	Stem	Tinosporacordifolia (Extract)	140 mg
2.	Mahalaxmivilasa Rasa	Processed	Classical Ayurveda preparation	75 mg
3.	Swaskaschintamani Rasa	Processed	Classical Ayurveda preparation	75 mg
4.	Jayamangal Rasa	Processed	Classical Ayurveda preparation	75 mg
5.	Yasadabhasma	Processed	Classical Ayurveda preparation	15 mg
6.	Triturated with the decoction of Vasa	Leaf	Adhatoda vasica	Q.S. (Quantum satis)

Svarnvir-IV tablet also contains incinerated mineral micro-powders, i.e., zinc bhasma, which has scientifically proven anti-viral agents [4-6]. From a chemical perspective, it may be predicted that this combination of anti-viral agents targets key viral molecules like 3CLpro, RdRp, spike protein, ACE2, helicase, and TMPRSSS2, which are involved in the pathogenesis of COVID-19. Another constituent, Swarnabhasma, is known for its immunity-boosting properties [5-7]. Thus, it may be presumed that this combination will not only correct the underlying pathology but also help in acquiring immunity that may be beneficial in the recurrence of the clinical symptoms as well [4-5]. The leaf juice of Vasa (*Adhatoda vasica*) is used for the trituration of all ingredients for making tablets and also has therapeutic activity against COVID-19 (Table 2). 

**Table 2:** The ingredients present in the Svarnvir-IV tablet (herbomineral formulation) include direct-acting antivirals and host-targeting antiviral agents; details are given with appropriate references Zn: Zinc, Au: Gold

Sl. No.	Name of the Ingredient	Common name in English	Important Phytoconstituents/Chemical constituent	Antiviral activity reference
1.	Yasada bhasma	Incinerated zinc compound	Zn (Zinc) nano-sized particles	Resveratrol-zinc nanoparticles or pterostilbene-zinc: potential COVID-19 monotherapy and adjuvant therapy [7].
2.	Guduchi satva (a water-soluble extract prepared by a pharmaceutical procedure)	Tinospora cordifolia	Cordifolioside A (1.62 μg/mg dry weight of GG), Magnoflorine (11.00 μg/mg), β-Ecdysone (1.75 μg/mg), and Palmatine (1.08 μg/mg)	Giloy Ghanvati (Tinospora cordifolia (Willd.) Hook. f. and Thomson) reversed the SARS-CoV-2 viral spike-protein-induced disease phenotype in the xenotransplant model of humanized zebrafish [8].
3.	Swarna bhasma (the common ingredient of all three herbomineral ingredients, i.e., Mahalaxmivilasa Rasa, Swasakasa Chintamani Rasa, and Jayamangala Rasa)	Incinerated gold compound	Au (gold) nano-sized particles	Gold, silver, copper, zinc, and iron oxide nanoparticles are effective against coronaviruses [4].
4.	Vasa	Adhatoda vasica	Decoction made with leaves used for the trituration of ingredients	Adhatoda Vasica attenuates inflammatory and hypoxic responses in preclinical mouse models: potential for repurposing in COVID-19-like conditions [9].

Objective

1) Determination of the CC50 (drug conc. that reduced cell viability by 50% when compared to the untreated group) and MNTD (maximum non-toxic dose) of the Svarnvir-IV tablet. 2) In vitro antiviral assessment of the Svarnvir-IV tablet-the formulation against the SARS-CoV-2 virus.

The in-vitro study was aimed at evaluating the Svarnvir-IV tablet for its potent protease inhibitor activity associated with antiviral activity against SARS-CoV-2. The study protocol was designed to first evaluate the cytotoxicity of the Svarnvir-IV tablet on Vero cells (Table 3). This was followed by virucidal activity assessment at two hours post-challenge and therapeutic activity assessment at 1 hour, 2 hours, and 4 hours post-treatment.

**Table 3:** Virucidal activity of Svarnvir-IV tablet against the test virus (Betacoronavirus-COVID-19) NCBI: National Center for Biotechnology Information

Test virus summary	Riboviria
Order	Nidovirales
Family	Coronaviridae
Genus	Betacoronavirus
Species	COVID-19
NCBI accession number for virus isolate	MT416726

To evaluate the anti-viral (anti-COVID-19) effect of the Svarnvir tablet, a cell-based assay was performed to reveal the cytotoxicity. Further, the determination of virucidal activity assessment (at 2 hours) and therapeutic activity assessment (at 1 hour, 2 hours, and 4 hours) were performed.

Cytotoxicity evaluation: determination of the CC50 and MNTD for the Svarnvir-IV tablet

Procedure for MNTD and CC50 determination: Six different dilutions of the test compound are added to the Vero cell monolayer in triplicate. The plates are incubated at 37°C in 5% CO2 for 72 hours. After incubation, a development solution (Minimum Essential Media [MEM] containing 2% fetal bovine serum as a negative control) is added to the cells, followed by incubation at 37°C in 5% CO2. Drug cytotoxicity, CC50, and MNTD are compared with that of 400 μmol formaldehyde as a positive control and diluent development solution (MEM) as a negative control. Absorbance is measured at the appropriate wavelength. MNTD and CC50 are derived.

In vitro antiviral assessment of Svarnvir-IV tablet against SARS-CoV-2 virus

A) Virucidal activity determination. B) Therapeutic activity determination

Virucidal activity determination

Herein, six different concentrations of the test drug below the MNTD value are assessed for antiviral potency in virucidal mode at the recommended time point in triplicate. Six different concentrations of the test compound in triplicate are incubated with the SARS-CoV-2 virus for 2 hours, recommended contact time. A virus-compound mixture is added to the Vero cell monolayer for infection. The plate is then incubated at 37°C in 5% CO2 for 72 hours. After incubation, the cytopathic effect is compared with hydroxychloroquine sulfate 0.72 μmol as a positive control and MEM as a negative control, and a quantitative CPE (cytopathic effect)-based score is made for EC50 (the compound concentration required to achieve 50% protection from virus) determination-induced cytopathogenicity. CPE stands for cytopathic effect, which is the damage caused to host cells by a virus during infection. When a virus infects a cell, it uses the host's cellular machinery to replicate itself, leading to the destruction of the cell and the release of new virus particles. CPE is a commonly used method for evaluating the antiviral activity of compounds or drugs. In this assay, cells are infected with the virus in the presence of the test compound, and the extent of virus-induced CPE is compared between treated and untreated cells. If the test compound has antiviral activity, it should reduce the severity of CPE compared to the untreated control. The significance of the CPE assay lies in its ability to provide a rapid and reliable measure of antiviral activity. It is relatively simple and inexpensive to perform and can be used to evaluate a wide range of viruses and test compounds. CPE can be visually observed and quantified using various methods, including microscopic examination, automated image analysis, and colorimetric assays.

Therapeutic activity determination

Six different concentrations of the test drug below the MNTD value are assessed for antiviral potency in therapeutic mode at recommended time points in triplicate. For each time point, Vero cells are infected with the SARS-CoV-2 virus for the recommended contact time points (1, 2, and 4 hours). Post-infection, six different concentrations of the test compound will be added to infected Vero cells in triplicate. The plate is incubated for 72 hours at 37°C in 5% CO2. After incubation, the cytopathic effect is compared with hydroxychloroquine sulfate 0.72 μmol as a positive control and MEM as a negative control, and a quantitative CPE-based score is made for EC50 determination.

Assessment of the drug in all modes will identify the antiviral potential of the drug candidate against SARS-CoV-2. EC50: compound concentration required to achieve 50% protection from virus-induced cytopathogenicity. CC50: compound concentration required to reduce cell viability by 50%. EC80: Drug concentration that gives 80% of Emax (maximum drug effect). SI* (selectivity index): ratio by CC50/EC50.

*The higher SI ratio = theoretically more effective and safer drug during in vivo treatment for a given viral infection. The EC50 value is the chemical concentration necessary to provide 50% protection against virus-induced cytopathogenicity. Following incubation, a quantitative CPE-based score will be generated for EC50 determination.

For determining the formulation's cytotoxicity, CC50, and MNTD, different dilutions of the test compound were tested on Vero cells and incubated at 37°C in 5% CO2 for 72 hours and compared with 400 µmol of formaldehyde as a positive control and diluent development solution MEM (Minimum Essential Media) as a negative control. Similarly, for virucidal activity, hydroxychloroquine sulfates 0.72 µmol as a positive control and MEM as a negative control were used, and other related details are given in Table 4.

**Table 4:** Details of experimental conditions for different protocols used in the present study. EC50: compound concentration required to achieve 50% protection from virus-induced cytopathogenicity, CC50: compound concentration required to reduce cell viability by 50%, EC80: drug concentration that gives 80% of Emax (maximum drug effect); SI* (selectivity index): ratio by CC50/EC50

Details of the drug samples' names			
Name of the drug molecule	Solubility	Appearance of the drug sample	Storage condition
Svarnvir-IV tablet	Water	Brown liquid	At room temperature
Details of the experimental conditions			
Host cell line used for testing	Vero cell line		
Virus used for testing	COVID-19 (SARS-CoV-2) at 0.1 Mol		
Test controls			
CC_50 _determination	400 μM Formaldehyde		
EC_50_ determination	0.72 μM Hydroxychloroquine Sulfate		
Diluent used for the assay	Minimum Essential Media (MEM) containing 2% Fetal Bovine Serum (FBS)		
Assay incubation period			
CC_50_	72 hours post addition of drug		
EC_50_/EC_80_	72 hours post-virus infection		
Concentration of the drug used for the assay	(Provided in the result section tables)		
Incubation conditions	37˚C, 5% CO_2_		
Software used for data analysis and interpretation	GraphPad Prism: Non-linear regression (curve fit)		

## Results

Svarnvir tablet, when incubated with SARS-CoV-2 virus at 0.1 multiplicity of infection (MoI) for 2 hours, exhibited virucidal activity against SARS-CoV-2 with an EC50 value of 0.0058 mg/ml. It also exhibited therapeutic activity when treated with the cells infected with the SARS-CoV-2 virus (0.1 MoI) for 1 hour, 2 hours, and 4 hours post-infection, with an EC50 value of 0.094mg/ml, 0.023 mg/ml, and 0.05 mg/ml, respectively.

The cytotoxicity research on the assessment of Svarnvir-IV reveals that at a dose of 0.500 mg/ml, the CC50 of the Svarnvir-IV tablet is not cytotoxic. The maximum non-toxic dosage, or MNTD, is not included in the statistics that are presented. After two hours, the Svarnvir-IV tablet's EC50 for virucidal activity was determined at a concentration of 0.0058 mg/ml. The EC50 values for therapeutic action were determined at various time intervals: 0.094 mg/ml after an hour, 0.023 mg/ml after two hours, and 0.050 mg/ml after four hours.

The selectivity index (SI), which is the ratio of CC50 to EC50, can be calculated as follows: For virucidal activity: SI = CC50/EC50 = 0.500/0.0058 = 86.2; for therapeutic activity after 1 hour: SI = CC50/EC50 = 0.500/0.094 = 5.3; for therapeutic activity after 2 hours: SI = CC50/EC50 = 0.500/0.023 = 21.7; for therapeutic activity after 4 hours: SI = CC50/EC50 = 0.500/0.050 = 10.0.

The Svarnvir-IV tablet is anticipated to be more efficient and safer when used in vivo to treat a specific viral infection with a higher SI ratio. Accordingly, the Svarnvir-IV tablet has the highest selectivity index for virucidal action based on the estimated SI values, with therapeutic activity peaking after two hours (Table 5).

**Table 5:** Details of the CC50, MNTD, and EC50 of the Svarnvir tablet CC50: compound concentration required to reduce cell viability by 50%. MNTD: maximum non-toxic dosage, EC50: compound concentration required to achieve 50% protection from virus-induced cytopathogenicity

Sample	CC50	MNTD	Mode of Assessment	Time point	EC50 mg/ml (milligram/milliliter)
Svarnvir-IV tablet	Not cytotoxic	0.500 mg/ml	Virucidal	2 hours	0.0058
			Therapeutic	1 hour	0.094
				2 hours	0.023
				4 hours	0.050

The data presented in Table 6 provides an evaluation of the Svarnvir-IV tablet's cytotoxicity after 72 hours of testing at various concentrations and their associated percent relative cell death. The percent relative cell death is 9.1% at a dose of 0.500 mg/ml, indicating that the medication is not cytotoxic at this level. Lower cytotoxicity is shown by a reduction in the percent of relative cell death at lower doses. The relative cell death rate is 4.3% at a concentration of 0.250 mg/ml, which is less than at a concentration of 0.500 mg/ml. At a dosage of 0.125 mg/ml, however, the relative cell death percentage is -14.7%, indicating cell growth as opposed to cell death. The percentage of relative cell death is negative or almost zero at even lower doses (0.025 mg/ml, 0.005 mg/ml, 0.001 mg/ml, and 0.0002 mg/ml), showing that the medication is not very cytotoxic at these levels. Svarnvir-IV tablet, in conclusion, seems to be non-cytotoxic at a concentration of 0.500 mg/ml, with decreasing cytotoxicity reported at lower doses, but more research would be needed to identify the ideal therapeutic dosage of the medication.

**Table 6:** Cytotoxicity assessment of Svarnvir-IV at 72 hours CC50: compound concentration required to reduce cell viability by 50%. MNTD: maximum non-toxic dosage.

Sample ID	Concentrations mg/ml	% Relative Cell Death Drug sample	MNTD mg/ml	CC50
Svarnvir-IV tablet	0.500	9.1	0.5	Non-cytotoxic
	0.250	4.3		
	0.125	-14.7		
	0.025	-9.7		
	0.005	-0.1		
	0.001	1.3		
	0.0002	2.2		

The antiviral evaluation of the Svarnvir-IV tablet against the SARS-CoV-2 virus in the virucidal mode is shown in Table 7, along with the doses that were tested and their associated relative percent cytopathic effect (CPE) inhibition. The Svarnvir-IV tablet's EC50, or the amount of medicine needed to produce 50% protection from virus-induced cytopathogenicity, is 0.0058 mg/ml when used in virucidal mode. Svarnvir-IV tablet exhibits 118.3% relative percent CPE inhibition at a dosage of 0.125 mg/ml, indicating antiviral activity. With a relative percent CPE inhibition of 51.1% at 0.025 mg/ml, 34.6% at 0.005 mg/ml, 32.4% at 0.001 mg/ml, and 28.9% at 0.0002 mg/ml, the antiviral activity diminishes as the drug concentration rises.

**Table 7:** Antiviral assessment of Svarnvir-IV tablets against the SARS-CoV-2 virus CPE: cytopathic effect

Sample ID	Concentrations mg/ml	Relative % CPE Inhibition Virucidal mode
Svarnvir-IV Tablet	0.125	118.3
	0.025	51.1
	0.005	34.6
	0.001	32.4
	0.0002	28.9

The maximum relative percent CPE inhibition was found at a dose of 0.125 mg/ml, indicating that the Svarnvir-IV tablet has antiviral action against the SARS-CoV-2 virus in the virucidal mode. The medication has an EC50 of 0.0058 mg/ml in the virucidal mode, demonstrating its effectiveness in obstructing virus-induced cytopathogenicity. To ascertain the drug's effectiveness in vivo and the ideal therapeutic concentration, more research would be necessary.

**Figure 1:**
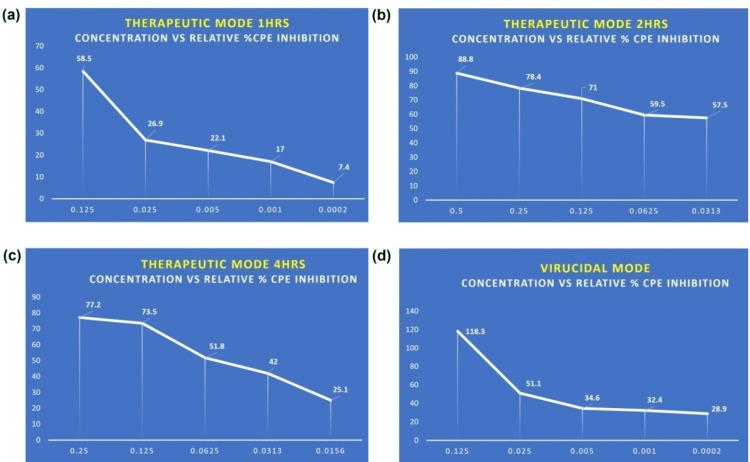
Schematic diagram showing Svarnvir tablet action on the SARS-COVID-19 virus. The schematic diagram (Figure 1) shows the Svarnvir tablet’s action on the SARS-COVID-19 virus. As per this investigation, it is found that the Svarnvir tablet is an inhibitor of the causative virus (SARS-CoV-2), based on the following results: (a) Antiviral assessment of the test sample against the SARS-CoV-2 virus (therapeutic mode). The maximum percent CPE inhibition was observed in the 0.125 mg/ml dose at 1 hour post-treatment. (b) Antiviral assessment of the test sample against SARS-CoV-2 virus (therapeutic mode). The maximum percent CPE inhibition of 88.8% was observed in the 0.5 mg/ml dose at 2 hours post-treatment. (c) Antiviral assessment of the test sample against the SARS-CoV-2 virus (therapeutic mode). The maximum percent CPE inhibition of 77.2% was observed in the 0.25 mg/ml dose at 4 hours post-treatment. (d) Antiviral assessment of the test sample against the SARS-CoV-2 virus (virucidal mode). The maximum percent CPE inhibition of 118.3% was observed in the 0.125 mg/ml dose at 2 hours of challenge with SAR-CoV-2.

Svarnvir-IV was also assessed for its antiviral activity against the SARS-CoV-2 virus in therapeutic mode. The relative percent cytopathic effect (CPE) inhibition at various sample concentrations after 1, 2, and 4 hours of treatment was used to gauge the sample's antiviral effectiveness (Table 8). In the present study, the sample had a relative percent CPE inhibition of 58.5% at a dose of 0.125 mg/ml after an hour of treatment; however, at a concentration of 0.5 mg/ml, it had a greater inhibition of 88.8%. Even at a dosage of 0.0156 mg/ml, the sample did not exhibit any appreciable inhibition. The relative percent CPE inhibition was seen to decline at decreasing sample concentrations, demonstrating a dose-dependent impact (Figure 1). For instance, the sample showed a relative percent CPE inhibition of 26.9% at a dosage of 0.025 mg/ml after one hour of treatment, which climbed to 78.4% after two hours and 77.2% after four hours of treatment.

**Table 8:** Antiviral assessment of Svarnvir-IV tablet 450 mg against SARS-CoV-2 virus in the therapeutic mode CPE: cytopathic effects inhibition

Sample ID	Antiviral assessment data (relative percent CPE)				
Svarnvir-IV Tablet	Concentrations mg/ml	Relative % CPE Inhibition Therapeutic Mode	Concentration mg/ml	Relative % CPE Inhibition Therapeutic Mode	
		1 hour		2 hour	4 hour
	0.125	58.5	0.5000	88.8	NP
	0.025	26.9	0.2500	78.4	77.2
	0.005	22.1	0.1250	71.0	73.5
	0.001	17.0	0.0625	59.5	51.8
	0.0002	7.4	0.0313	57.5	42.0
			0.0156	NP	25.1

Overall, the evidence points to the Svarnvir-IV tablet having antiviral action against the SARS-CoV-2 virus in a therapeutic mode, with the concentration and length of therapy being factors in the reduction of viral reproduction. However, more research is required to ascertain the sample's effectiveness and safety in clinical settings. 

## Discussion

The development and production of broad-spectrum antiviral medications not only combat COVID-19 but also builds arsenals for future viral epidemics. The richness of Ayurvedic information on virus-host interactions that has been and continues to be acquired will guide and stimulate the development of host-targeting antivirals. These offer the potential benefits of broad-spectrum therapeutic action and viral evasion resistance. Antiviral drug development, in general, will benefit from the increased social knowledge of Ayurvedic medicine and the COVID-19 outbreak, therefore protecting humanity from future viral epidemics. It is found that the antiviral effects of ingredients present in the Svarnvir-IV tablet (450 mg) will block the cell cycle protein of the pathogen and increase innate host immunity. The Svarnvir-IV tablet contains gold microparticles (like nanoparticles) in the absorbable form that inhibit early stages of viral replication, viral entry, and viral attachment by penetrating cells [5], and the sublingual route of gold bhasma administration showed maximum bioavailability [6], and a zinc compound that inhibits viral replication through modulation of the glutathione redox system [7].

Endoplasmic reticulum (ER) stress and unfolded protein response (UPR) activation are known to play important roles in viral replication and pathogenesis during coronavirus infection [10]. To sustain protein folding, coronavirus infection stimulates the expression of the ER protein folding chaperons GRP78, GRP94, and other ER stress-related genes [11]. UPR activation is significant in cells overexpressing the SARS-CoV-2 spike protein and other viral proteins [12-13]. Thus, auranofin's suppression of redox enzymes, such as thioredoxin reductase, and the development of ER stress might have a considerable impact on SARS-CoV-2 protein production [14].

The current study was conducted to evaluate the antiviral activity of the Svarnvir-IV tablet against the SARS-CoV-2 virus in vitro. Svarnvir-IV tablet, when incubated with SARS-CoV-2 virus at 0.1 multiplicity of infection (MoI) for 2 hours, exhibited virucidal activity against SARS-CoV-2 with an EC50 value of 0.0058 mg/ml. It also exhibited therapeutic activity when treated with cells infected with the SARS-CoV-2 virus (0.1 MoI) for 1 hour, 2 hours, and 4 hours post-infection, with an EC50 value of 0.094mg/ml, 0.023 mg/ml, and 0.05 mg/ml, respectively. Given the in vitro effectiveness demonstrated in the study, the Svarnvir-IV tablet should be studied further for its potential as an orally administered medication for SARS-CoV-2 therapeutic uses. The original supporting data obtained from this study, along with existing Ayurvedic traditional information, will help to understand the potential of Svarnvir-IV tablets in the treatment of COVID-19.

Limitations: The Svarnvir-IV tablet's antiviral activity was assessed in vitro, but more research is needed to determine its safety and effectiveness in both animal models and humans. To fully comprehend the precise mode of action of this medicine, more investigation is also required.

## Conclusions

Direct-acting antivirals (DAAs) are being created at a rapid rate, and there is a great deal of anticipation that these efforts will result in very effective Ayurvedic antiviral medications. As per this investigation, it is found that the Svarnvir-IV tablet is an inhibitor of the causative virus (SARS-CoV-2) based on the above results. Svarnvir-IV tablet, when incubated with SARS-CoV-2 virus at 0.1 multiplicity of infection (MoI) for 2 hours, exhibited virucidal activity against SARS-CoV-2 with an EC50 value of 0.0058 mg/ml. It also exhibited therapeutic activity when treated with cells infected with the SARS-CoV-2 virus (0.1 MoI) for 1 hour, 2 hours, and 4 hours post-infection, with an EC50 value of 0.094mg/ml, 0.023 mg/ml, and 0.05 mg/ml, respectively. The original supporting data obtained from this study, along with existing Ayurvedic traditional information, will help to understand the potential of the Svarnvir-IV tablet in the treatment of COVID-19. The drug can be rapidly repurposed for safe and highly efficacious clinic applications for treating COVID-19 patients.
